# Classification of a Violacein-Producing Psychrophilic Group of Isolates Associated with Freshwater in Antarctica and Description of *Rugamonas violacea* sp. nov

**DOI:** 10.1128/spectrum.00452-21

**Published:** 2021-08-11

**Authors:** Ivo Sedláček, Pavla Holochová, Roman Sobotka, Hans-Jürgen Busse, Pavel Švec, Stanislava Králová, Ondrej Šedo, Jan Pilný, Eva Staňková, Vendula Koublová, Karel Sedlář

**Affiliations:** a Department of Experimental Biology, Czech Collection of Microorganisms, Faculty of Science, Masaryk University, Brno, Czech Republic; b Centrum Algatech, MBÚ AV ČR, Třeboň, Czech Republic; c Institut für Mikrobiologie, Veterinärmedizinische Universität Wien, Vienna, Austria; d Central European Institute of Technology, Masaryk University, Brno, Czech Republic; e Department of Biomedical Engineering, Faculty of Electrical Engineering and Communication, Brno University of Technology, Brno, Czech Republic; University of Minnesota

**Keywords:** psychrophiles, *Rugamonas*, taxonomy, Antarctica, violacein, species description

## Abstract

A group of 11 bacterial strains was isolated from streams and lakes located in a deglaciated northern part of James Ross Island, Antarctica. They were rod-shaped, Gram-stain-negative, motile, and catalase-positive and produced blue-violet-pigmented colonies on R2A agar. A polyphasic taxonomic approach based on 16S rRNA gene sequencing, whole-genome sequencing, automated ribotyping, repetitive element sequence-based PCR (rep-PCR), MALDI-TOF MS, fatty acid profile, chemotaxonomy analyses, and extensive biotyping was applied in order to clarify the taxonomic position of these isolates. Phylogenetic analysis based on the 16S rRNA gene indicated that all the isolates constituted a coherent group belonging to the genus *Rugamonas*. The closest relatives to the representative isolate P5900^T^ were Rugamonas rubra CCM 3730^T^, *Rugamonas rivuli* FT103W^T^, and *Rugamonas aquatica* FT29W^T^, exhibiting 99.2%, 99.1%, and 98.6% 16S rRNA pairwise similarity, respectively. The average nucleotide identity and digital DNA-DNA hybridization values calculated from the whole-genome sequencing data clearly proved that P5900^T^ represents a distinct *Rugamonas* species. The G+C content of genomic DNAs was 66.1 mol%. The major components in fatty acid profiles were summed feature 3 (C_16:1_ *ω7c*/C_16:1_ *ω6c*), C _16:0_, and C_12:0_. The cellular quinone content contained exclusively ubiquinone Q-8. The predominant polar lipids were diphosphatidylglycerol, phosphatidylglycerol, and phosphatidylethanolamine. The polyamine pattern was composed of putrescine, 2-hydroxputrescine, and spermidine.

**IMPORTANCE** Our polyphasic approach provides a new understanding of the taxonomy of novel pigmented *Rugamonas* species isolated from freshwater samples in Antarctica. The isolates showed considerable extracellular bactericidal secretions. The antagonistic activity of studied isolates against selected pathogens was proved by this study and implied the importance of such compounds’ production among aquatic bacteria. The psychrophilic and violacein-producing species *Roseomonas violacea* may play a role in the diverse consortium among pigmented bacteria in the Antarctic water environment. Based on all the obtained results, we propose a novel species for which the name *Rugamonas violacea* sp. nov. is suggested, with the type strain P5900^T^ (CCM 8940^T^; LMG 32105^T^). Isolates of *R. violacea* were obtained from different aquatic localities, and they represent the autochthonous part of the water microbiome in Antarctica.

## INTRODUCTION

The genus *Rugamonas* with the species Rugamonas rubra was described more than 30 years ago (1) and comprises red-pigmented bacterial strains isolated from river water. At present, the genus *Rugamonas* is a member of the family *Oxalobacteraceae*, class *Betaproteobacteria*, and phylum *Proteobacteria*. There are only three species with validly published names in the genus *Rugamonas* (http://lpsn.dsmz.de/genus/rugamonas) (2), and these species are associated with aquatic environments. Until recently, the species R. rubra was the sole taxon of the genus; however, two new species, Rugamonas aquatica and Rugamonas rivuli, both from a subtropical stream, were described recently (3). Both these novel species have light brown colonies.

Here, we characterize purple-pigmented isolates which were retrieved from various freshwater sources in Antarctica over the period of 2013 to 2019. The purple pigment production facilitates their isolation from primocultures, but this feature is not taxonomically relevant, as other Gram-stain-negative species produce purple or violet pigments, such as *Iodobacter* spp. (4, 5), *Massilia* spp. (6, 7), *Chromobacterium* spp. (8–12), and Pseudoduganella violaceinigra (13). Violet-pigmented bacteria have insecticidal properties (10), and the microbial antibiotic pigment violacein exhibits several biological activities, such as antiviral, bactericidal, tumoricidal, trypanocidal, and antileishmanial activities (14–16). Potential applications of violacein have gained increasing importance in industrial markets, such as in medicine, cosmetics, and textiles (17).

In this study, a group of violet-pigmented isolates was selected from oligotrophic strains obtained within the framework of a project focused on psychrophilic environmental bacteria from Antarctica and analyzed by 16S rRNA sequencing. Subsequently, a small group of 11 violet-pigmented isolates was characterized by the polyphasic approach.

## TAXONOMY

### Description of *Rugamonas violacea* sp. nov.

**FIG 1:**
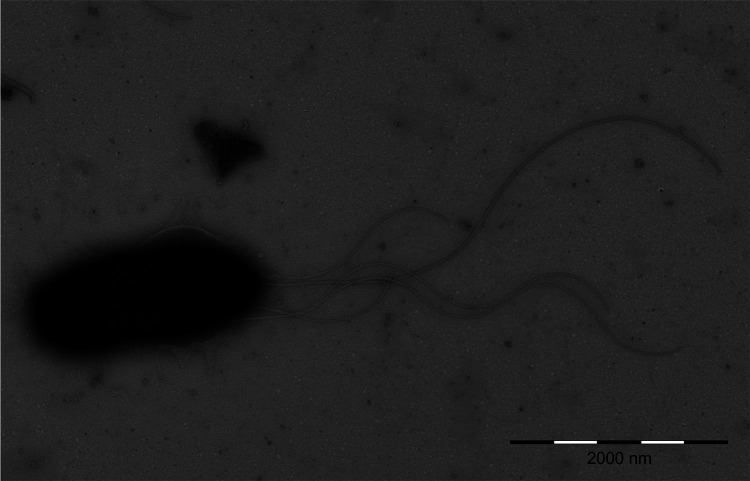
Cell morphology of strain P5900^T^ observed using transmission electron microscopy performed with a Philips Morgagni 268D electron microscope (FEI Company, Czech Republic). Negative staining with 2% ammonium molybdate. Bar represents 2,000 nm (original magnification ×12,000).

*R. violacea* etymology: vi.o.la'ce.a. L. fem. adj. *violacea* of violet, relating to the colony color. The description of the species is based on 11 strains. Cells are Gram-stain-negative non-spore-forming shorter rods, occurring predominantly separately or in irregular clusters and motile with a bundle of polar flagella (Fig. 1). Cell width ranged from 680 nm to 963 nm and length from 1.4 μm to 2 μm. Colonies on R2A medium are circular, whole margin, convex, smooth, glistening with bold blue-violet endopigment violacein, approximately 2 mm in diameter after 3 days cultivation at 20°C, and moderately slimy with strong adherence to agar surface. Aerobic growth occurs only on R2A agar and PCA agar at 20°C. Good growth is observed between 1°C and 25°C, in the pH range of 6.0 to 9.0, and in the presence of 0.5% NaCl (wt/vol). Acid production (aerobic) from glucose, fructose, mannitol, and maltose, while fermentation of glucose in oxidative/fermentation (OF) test medium is negative. Catalase, alkaline phosphatase, acid phosphatase, leucine arylamidase, valine arylamidase (weak), α-chymotrypsin, and naphthol-AS-BI-phosphohydrolase positive by API ZYM. Malonate utilization positive. Gelatin, casein, esculin, and tyrosine hydrolysis positive. Nitrate reduction, nitrite reduction, Voges-Proskauer test (acetoin), and hydrolysis of lecithin positive for most strains. Esterase (C4), esterase lipase (C8), lipase (C14), cystine arylamidase, trypsin, α-galactosidase, β-galactosidase, β-glucuronidase, β-glucosidase, *N*-acetyl-β-glucosaminidase, α-mannosidase, and α-fucosidase negative by API ZYM. Oxidase, fluorescein (King B medium), urease, H_2_S production, methyl-red test, lysine and ornithine decarboxylase, arginine dihydrolase, Simmons citrate, and acetamide utilization negative. Hydrolysis of Tween 80, *o*-nitrophenyl-β-d-galactopyranoside (ONPG), starch, and DNA negative. Variable phenotypic reactions of *R. violacea* strains are listed in Table 1. Resistant to ampicillin, carbenicillin, ceftazidime, and chloramphenicol, but sensitive to ciprofloxacin, gentamicin, imipenem, kanamycin, co-trimoxazole, streptomycin, and tetracycline. Sensitivity or resistance to piperacillin was strain-dependent (Table 1).

**TABLE 1:** Variable reactions of *Rugamonas violacea* sp. nov. strains^*a*^

Strain no.	Growth in 1% NaCl	Growth at pH 5	Nitrate reduction	V-P test (acetoin)	Nitrite reduction	Hydrolysis of lecithin	Acid from xylose	α-Glucosidase	Piperacillin
P4871	w	−	+	+	+	+	−	w	S
P5042	−	+	+	w	+	−	−	−	R
P5460	+	−	+	+	+	+	−	w	S
P5807	w	−	+	+	+	+	+	−	S
P5900^T^	w	−	−	+	+	+	−	−	S
P5997	+	−	+	−	+	+	−	w	R
P6607	w	+	+	+	+	+	+	+	S
P7310	w	+	+	+	+	+	+	w	R
P7476	w	−	+	−	+	+	−	−	S
P8911	−	+	+	w	−	+	+	w	S
P11744	−	−	+	+	−	+	−	−	R

a +, positive; w, weakly positive; −, negative; S, sensitive; R, resistant.

All strains were positive for the utilization (Biolog) of d-maltose, d-trehalose, d-cellobiose, sucrose, *N*-acetyl-d-galactosamine, d-fructose, d-mannitol, glycerol, d-glucose-6-phosphate, gelatin, l-aspartic acid, l-glutamic acid, l-histidine, d-gluconic acid, l-malic acid, and Tween 40 as carbon sources and negative for the utilization of gentiobiose, stachyose, d-raffinose, α-d-lactose, d-melibiose, β-methyl-d-glucoside, d-salicin, *N*-acetyl-d-glucosamine, *N*-acetyl-β-d-mannosamine, *N*-acetyl neuraminic acid, 3-methyl glucose, d-fucose, l-fucose, l-rhamnose, d-sorbitol, d-aspartic acid, d-serine, l-pyroglutamic acid, d-galacturonic acid, d-galactonic acid lactone, d-glucuronic acid, glucuronamide, mucic acid, quinic acid, d-saccharic acid, p-hydroxyphenylacetic acid, d-lactic acid methyl ester, α-keto glutaric acid, d-malic acid, γ-amino-butyric acid, α-hydroxy-butyric acid, acetoacetic acid, and formic acid. Variable results of *Rugamonas violacea* strains obtained in the Biolog GEN III MicroPlate are shown in Table 2.

**TABLE 2:** Variable reactions of *Rugamonas violacea* sp. nov. strains on Biolog GEN III MicroPlate^*a*^

Test	Result for strain:										
	P4871	P5042	P5460	P5807	P5900^T^	P5997	P6607	P7310	P7476	P8911	P11744
Dextrin	b	+	+	+	−	b	+	−	+	−	−
d-turanose	−	−	b	+	+	−	b	+	b	+	−
α-glucose	+	+	+	−	−	+	+	+	+	+	+
d-mannose	−	−	+	−	+	+	+	+	b	+	+
d-galactose	b	+	−	+	+	b	b	+	b	+	+
Inosine	−	+	+	+	+	−	+	−	+	−	−
d-arabitol	+	+	+	−	−	+	−	+	+	−	+
myo-inositol	b	−	−	−	−	b	−	+	+	+	+
d-fructose-6-PO4	−	+	−	+	−	−	+	−	−	+	−
Glycyl-l-proline	−	−	−	−	−	−	+	+	−	−	−
l-alanine	−	−	−	−	−	−	+	−	+	−	−
l-arginine	−	−	b	−	−	b	b	−	b	−	−
l-serine	+	−	−	−	−	−	−	−	b	−	−
Pectin	+	+	b	b	+	+	b	+	+	−	+
Methyl pyruvate	b	−	−	+	+	b	−	+	+	+	+
l-lactic acid	−	−	−	+	+	b	b	+	b	w	+
Citric acid	−	−	+	−	−	−	+	−	−	−	−
Bromo-succinic acid	+	−	−	−	−	−	−	−	b	+	+
β-OH-d,l-butyric acid	+	−	−	+	+	+	+	+	+	+	w
α-keto butyric acid	w	−	−	−	−	−	−	+	−	+	+
Propionic acid	−	−	−	b	−	−	b	+	b	+	+
Acetic acid	−	−	−	−	−	−	+	+	b	+	−

a All data were taken from this study using two replications. +, positive; w, weak; b, borderline; −, negative.

Type strain is P5900^T^ (CCM 8940^T^; LMG 32105^T^). The DNA G+C content of strain P5900^T^ is 66.1 mol%. Almost all characteristics of the type strain P5900^T^ are in agreement with the species description. The strain-dependent test results of P5900^T^ are presented in Tables 1 and 2.

## RESULTS AND DISCUSSION

### Bacterial strain selection.

**FIG 2:**
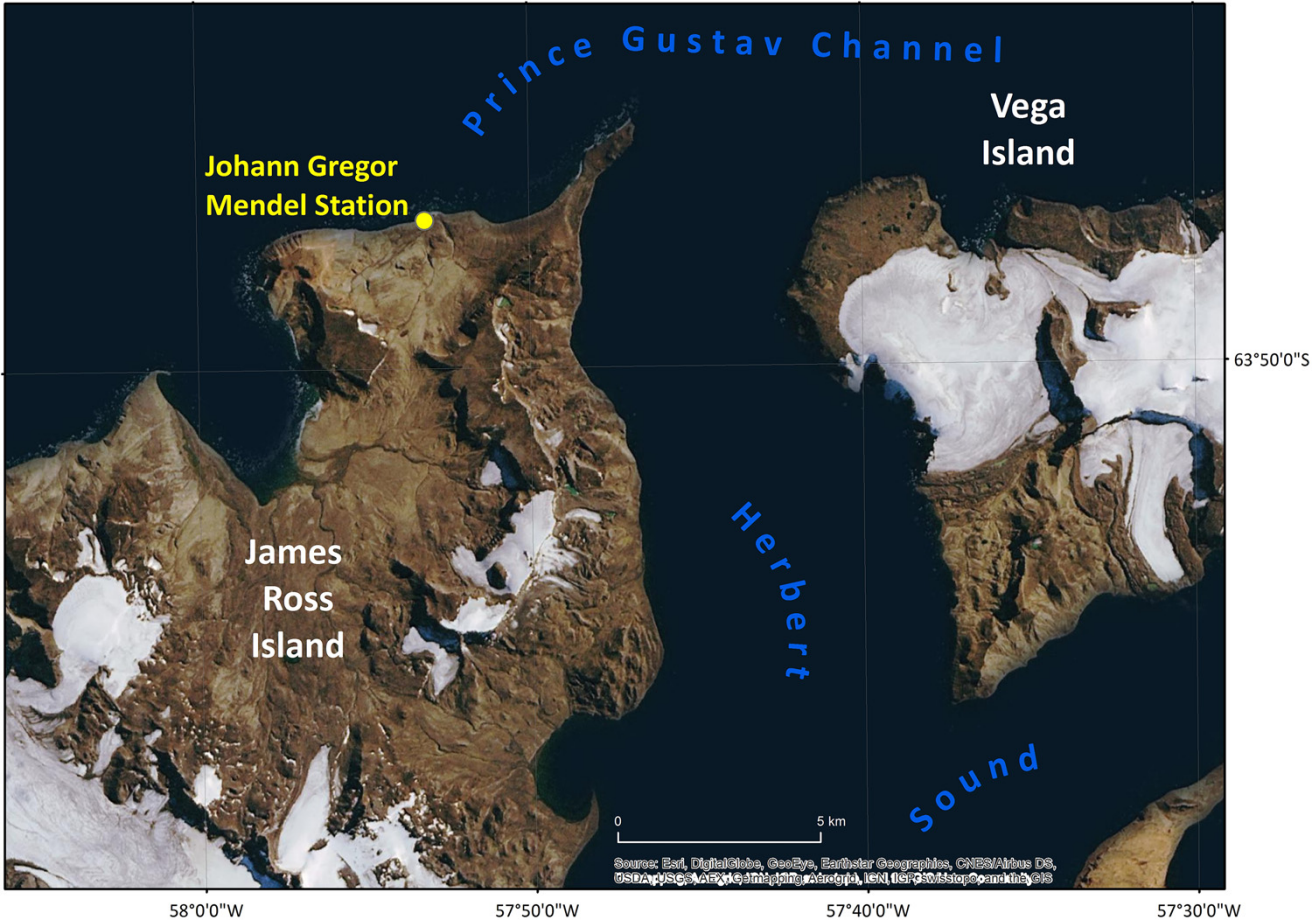
Sampling area in a deglaciated northern part of James Ross Island and Vega Island, Antarctica.

A total of 2,000 strains were isolated within the framework of a project focused on psychrophilic bacteria from freshwater sources in Antarctica. The sampling sites were situated in a deglaciated northern part of James Ross Island and Vega Island, Antarctica (Table 3 and Fig. 2). The dark violet pigment was produced by 24 isolates, and a small group of 11 pigmented isolates were nearly identical in their 16S rRNA gene sequence analysis (Table 4). These strains were characterized further in this study.

**TABLE 3:** Source of *Rugamonas violacea* sp. nov. strains

Strain no.	CCM no.	Year of isolation	Locality	GPS coordinates	Original no.
P4871		2013	Marshland between small and big Lachman lakes, James Ross Island	57°48′42″W 63°47′53″S	13 V 22/6
P5042		2013	Spring below plateau Berry Hill, James Ross Island	57°51′10″W 63°48′26″S	13 V 2/1
P5460		2014	Dirty stream, upper part, James Ross Island	57°53′47″W 63°49′31″S	14 V 10/1
P5807		2014	Solorina valley, glacial stream, lower part, James Ross Island	57°48′16″W 63°53′15″S	14 V 54/3
P5900^T^	8940^T^	2014	Solorina valley, small brook, lower part, James Ross Island	57°46′48″W 63°53′36″S	14 V 55/6
P5997		2014	Algal stream, lower part, James Ross Island	57°52′36″W 63°48′19″S	14 V 14/7
P6607		2015	Active layer of permafrost, nearby J.G. Mendel station, James Ross Island	57°53′20″W 63°48′15″S	Permafrost 1/8 (melted)
P7310	9027	2016	Spring from snowfield, Panorama Pass, James Ross Island	57°50′29″W 63°48′50″S	16 V 15/1
P7476		2016	Marshland nearby Dirty stream, James Ross Island	57°53′09″W 63°48′38″S	16 V 46/4
P8911		2017	Stream nearby sea coast, Dinn Cliffs, James Ross Island	57°51′55″W 63°58′12″S	17 V 175/3
P11744	9026	2019	Water with sediment, lake Ultra Green II; Vega Island	57°35′42″W 63°52′40″S	19 S 21/4

**TABLE 4:** Pairwise 16S rRNA gene sequence similarity values (%) of the *Rugamonas violacea* sp. nov. strains; all data were taken from this study

Strain no.	16S contents (%) of strain no.:										
	P4871	P5042	P7476	P11744	P5997	P7310	P5460	P5807	P5900^T^	P6607	P8911
P4871	100										
P5042	100	100									
P7476	100	100	100								
P11744	100	100	100	100							
P5997	99.9	99.9	99.9	99.9	100						
P7310	99.8	99.8	99.8	99.8	99.9	100					
P5460	99.8	99.8	99.8	99.8	99.8	99.9	100				
P5807	99.8	99.8	99.8	99.8	99.8	99.9	100	100			
P5900^T^	99.8	99.8	99.8	99.8	99.8	99.9	100	100	100		
P6607	99.8	99.8	99.8	99.8	99.8	99.9	100	100	100	100	
P8911	99.8	99.8	99.8	99.8	99.8	99.99	100	100	100	100	100

### Phylogenetic relationship based on 16S rRNA gene sequencing.

**FIG 3:**
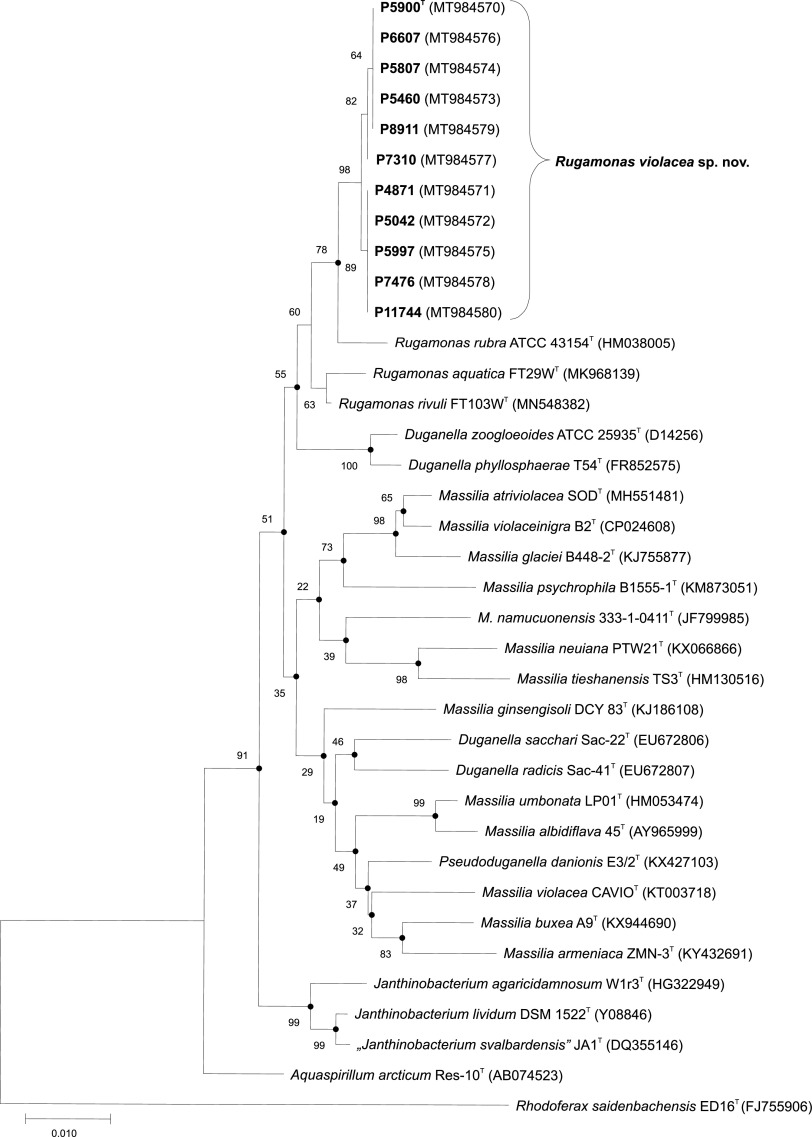
Unrooted neighbor‐joining tree based on 16S rRNA gene sequence comparisons showing the phylogenetic position of Antarctic *Rugamonas* isolates classified to *Rugamonas violacea*. Bootstrap probability values (percentages of 1,000 tree replications) greater than 50% are shown at branch points. The evolutionary distances are in the number of base substitutions per site. All ambiguous positions were removed for each sequence pair. Filled circles indicate that the corresponding nodes are also obtained in the maximum‐likelihood tree. Rhodoferax saidenbachensis ED16 was used as an outgroup. GenBank accession numbers of 16S rRNA genes sequences are given in parentheses. Bar, 0.010 substitutions per nucleotide position.

Each of the 11 violet-pigmented Antarctic environmental isolates (Table 3) was initially identified by 16S rRNA gene sequencing. The 16S rRNA gene sequence similarities among the analyzed set of strains varied from 99.8% to 100% and initially placed all the strains within the genus *Rugamonas*. The closest relative species identified by pairwise sequence alignment (Table 5) were *R. rubra* (99.2% similarity), *R. rivuli* (99.1 to 99.3%), and *R. aquatica* (98.6 to 98.8%). The phylogenetic reconstruction based on 16S rRNA performed using the maximum-likelihood (ML) and neighbor-joining (NJ) methods grouped all the analyzed strains into a common clade with 98% bootstrap support (Fig. 3). The majority of the internal branches of the reconstructed ML tree were identical to those in the NJ tree. These results clearly showed that the analyzed set of strains belongs to the genus *Rugamonas*. The GenBank/EMBL/DDBJ accession number for the complete 16S rRNA gene sequence of strain P5900^T^ is MT984570. The GenBank/EMBL/DDBJ accession numbers for the partial 16S rRNA gene sequences of other pigmented *Rugamonas* analyzed isolates are MT984571 to MT984580 and are listed in Fig. 3. However, the 16S rRNA gene sequence similarities between the isolates and the reference strains were above the threshold value of 98.7% suggested for species delineation (18) and did not allow species differentiation within the set of strains. Therefore, the average nucleotide identity (ANI) analysis was performed to characterize the taxonomic position of strain P5900^T^ in more detail.

**TABLE 5:** Pairwise 16S rRNA gene sequence similarity values (%) of the analyzed genes of *Rugamonas violacea* sp. nov. representative strains and reference types

Representative strains	Gene sequence similarity value (%) for reference strain:					
	*R. violacea* P5900^T^	*R. violacea* P11744	*R. violacea* P7310	*R. rubra* CCM 3730^T^	*R. rivuli* FT103W^T^	*R. aquatica* FT29W^T^
*R. violacea* P5900^T^	100	99.8	99.9	99.2	99.1	98.6
*R. violacea* P11744	99.8	100	99.9	99.2	99.3	98.8
*R. violacea* P7310	99.9	99.8	100	99.2	99.2	98.7

### Basic genome characterization and average nucleotide identity.

The genome accession number of P5900^T^ (CCM 8940^T^) in the GCM type strains genome database is GCM60018930 (http://gctype.wdcm.org/sequencing/GCM10018930). The Whole-Genome Shotgun project of *Rugamonas violacea* P5900^T^ has been deposited at GenBank/EMBL/DDBJ under the accession no. JAEMNU000000000, and the version described in this paper is JAEMNU010000000. The resulting draft genome size was 6.9 Mb with 280 contigs (*N*_50_ = 53,085; *L*_50_ = 40). The annotation revealed 5,998 genes divided into 3,696 operons with 5,805 protein-coding genes (CDS) and 95 RNA genes, including 8 genes for rRNAs (5 5S, 2 16S, and 1 23S rRNA) and 83 genes for tRNAs. The genomic G+C content was 66.1 mol%, which clearly corresponds with the percentage values described for other *Rugamonas* spp. (1, 2). Functional annotation of CDS in the form of division into clusters of orthologous genes (COG) assigned the majority of CDS (3,936 genes, almost 68% of all CDS) to a particular function, while the remaining genes were those with an unassigned group or the “unknown function” category (group S), representing 852 and 1,017 genes, respectively, suggesting that future experiments with the strain may reveal novel hitherto unreported properties (Table S1a). The other most abundant group was (K) “transcription,” with 428 genes, and many others were classified into basic functions, such as 416 genes in (group E) “amino acid transport and metabolism,” 357 in (group M) “cell wall/membrane/envelope biogenesis,” and 324 in (group T) “signal transduction mechanisms,” together with 284 genes in (group C) “energy production and conversion,” 252 in (group N) “cell motility,” and 248 in (group P) “inorganic ion transport and metabolism,” and results confirmed the psychrophilic nature of the strain and its ability to adapt to extreme environments. No gene was associated with class (X) “mobilome: prophages, transposons,” and only a single incomplete phage was predicted in the 20th contig (JAEMNU010000020) within the region 12,025 to 31,927 containing 19 viral genes out of 24 genes in this region. This lack of foreign genetic material could be a sign of an active bacterial immune system. Three CRISPR (clustered regularly interspaced short palindromic repeat) arrays were found in the *R. violacea* P5900^T^ genome (Table S1b). It is known that a CRISPR-associated system (Cas) can form an adaptive immune system in bacteria (19). The size of these three arrays ranged from 259 to 3,876 bp and 3 to 58 spacer units. Nevertheless, none of these arrays had any known *cas* or *cas*-like genes in their neighborhoods.

Since the isolate was pigmented and formed slimy capsules, we focused on an analysis of the genes contributing to violacein production and survival under adverse climatic conditions. Operon prediction and pathway mapping revealed the presence of the whole *vioABCDE* operon. Proteins related to violacein biosynthesis were identified as vioA (MBJ7311990.1), vioB (MBJ7311991.1), vioC (MBJ7311992.1), vioD (MBJ7311993.1), and vioE (MBJ7311994.1). Moreover, additional proteins known to regulate violacein biosynthesis were found: the sensor kinase jqsS (MBJ7309130.1) and the response regulator jqsR (MBJ7309129.1) coded by a single operon or autoinducer synthase jqsA (MBJ7309131.1). Since the upstream region of the *vioABCDE* operon contained a possible regulatory motif with more than 85% similarity to the known jqsR motif TTGA_N6/7_TCAA (20), we believe that the expression of the violacein biosynthesis operon could be dependent on quorum sensing in *Rugamonas violacea*. Further exploration of genes assigned to the functional group (N) “cell motility” revealed 84 KEGG orthologues belonging to the KEGG BRITE group ko02035 “bacterial motility proteins.” While 53 of them corresponded to the flagellar system, containing all the necessary genes for flagellar assembly, the remaining 31 orthologues corresponded to the pilus system. Although no perfect hits of CDS against the comprehensive antibiotic resistance database (CARD) were found, 13 genes with sequence similarity of >55% to and length 100% ± 5% of known antibiotics resistance genes were detected (Table S2). Three different mechanisms of resistance (antibiotic target alteration, antibiotic efflux, and antibiotic inactivation) were found, while efflux was the most prevalent one, with 6 genes.

Average nucleotide identity (ANI) values were determined, and the intergenomic distances between the genome sequences of strain P5900^T^ and reference type strains of *Rugamonas* spp. were analyzed. The ANI values between P5900^T^ and *R. rubra* ATCC 43154^T^, *R. rivuli* FT103W^T^, and *R. aquatica* FT29W^T^ were 91.6%, 81.4%, and 81.1%, respectively, and the digital DNA-DNA hybridization (dDDH) values between P5900^T^ and *R. rubra* ATCC 43154^T^, *R. rivuli* FT103W^T^, and *R. aquatica* FT29W^T^ were 44.9%, 24.9%, and 24.7%, respectively. These ANI and dDDH values obtained between the analyzed strain P5900^T^ and *Rugamonas* spp. types were far below the thresholds of 95 to 96% (ANI) and 70% (dDDH) for differentiating bacterial species (18, 21, 22). These results confirmed that strain P5900^T^ represents a distinct *Rugamonas* species.

### MALDI-TOF MS.

**FIG 4:**
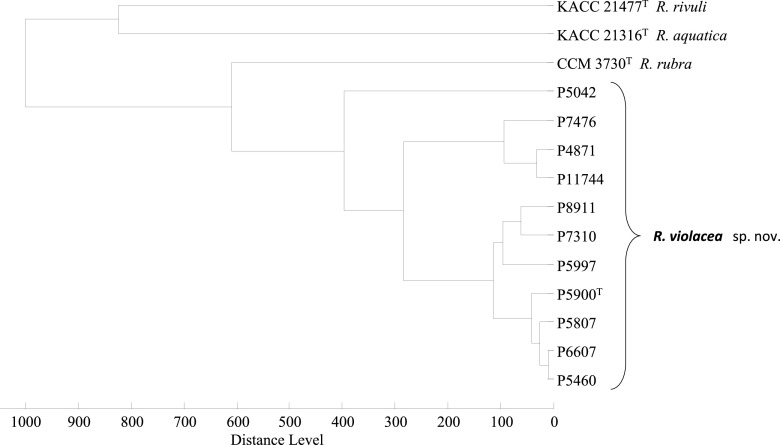
Dendrogram obtained by cluster analysis of MALDI-TOF mass spectra of *R. violacea* sp. nov. strains and closely related *Rugamonas* species type strains constructed by Biotyper 3.0 (Bruker Daltonics) software using the Pearson’s product moment coefficient as a measure of similarity and the unweighted pair group average linked method (UPGMA) as a grouping method. Distance is displayed in relative units.

The analyzed isolates were clearly distinguished from reference types of *Rugamonas* spp. by means of matrix-assisted laser desorption ionization–time of flight (MALDI-TOF) mass spectrometric protein fingerprinting, as all isolates were grouped into one cluster in the dendrogram (Fig. 4). While all the isolates shared 18 signals, only 4 of these signals (*m/z *= 3,232, 3,592, 7,182, and 8,417) were not observed in the mass spectra of any of the other three type strains. The isolates were further subclustered into two groups due to a few different signals in the *m/z* range 4,000 to 5,000. The current MALDI Biotyper database (version 9.0.0.0 involving 8,468 entries) does not cover any data of validly published *Rugamonas* species, and thus identification to the species/genera level was not possible. However, after the database was extended with the data of the three type strains and P5900^T^, all isolates were assigned to P5900^T^ with a Biotyper log (score) greater than 2.300, while 5 of them were also assigned to Rugamonas rubra CCM 3735^T^ with scores greater than 2.000.

### Genotyping by RiboPrinter and rep-PCR.

Numerical analysis of the obtained ribotypes clustered the analyzed isolates into several subclusters with correlation similarities of around 90% (Fig. S1a). The closest ribotype patterns were revealed between the pair of isolates P5807 and P5900^T^ (98% similarity), which were obtained from different water sources, which shows that they are not identical. On the other hand, isolate P7476, which was isolated from marshland, gave the most distant fingerprints. The obtained ribotype patterns separated the investigated pigmented isolates from each other, as can be clearly seen from the dendrogram. The repetitive element sequence-based PCR (rep-PCR) analysis showed an allied band pattern among the studied isolates (Fig. S1b), and isolates were clustered in three subclusters. The majority of rep-PCR subclusters are in conformity with ribotyping clustering results, because subclusters have an identical composition or almost the same isolates in the subclusters. These genotyping results together with the different collection localities demonstrated the nonclonal relationship of the analyzed isolates.

### Chemotaxonomic characterization.

The predominant fatty acids were summed feature 3 (C_16:1_ *ω7c*/C_16:1_ *ω6c*), C_16:0_, C_12:0_, summed feature 8 (C_18:1_ *ω7c*/iso-C_18:1_ *ω6c*), and C_10:0_ 3OH. The complete cellular fatty acids composition of the analyzed 11 strains is given in Table 6. For comparison purposes, the cellular fatty acids content of the phylogenetically closest *Rugamonas* spp. as well as other violacein-producing species was analyzed and is included in Table 6. The fatty acid profile of newly described species is qualitatively and quantitatively similar to *R. rivuli* and *R. rubra* tested in parallel and thus supports its classification to the genus *Rugamonas*. Fatty acid profiles enable only differentiation between *R. violacea* sp. nov. and *R. rivuli* KACC 21477^T^ based on presence of C_17:0_ cyclo. Two other validly named *Rugamonas* species, *R. rubra* and *R. aquatica*, show only minor qualitative differences presented in Table 6 and described by Lu et al. (3). The absence of C_12:0_ 2OH, C_12:0_ 3OH, iso-C_16:0_, and C_17:0_ cyclo among the analyzed strains clearly distinguished those from violacein-producing species Chromobacterium violaceum, Janthinobacterium lividum, or Iodobacter fluviatile (Table 6).

**TABLE 6:** Cellular fatty acid contents (%) of *Rugamonas violacea* sp. nov. strains and reference cultures^*a*^

Fatty acid	Value for strain no.:						
	P5900^T^	P4871, P5042, P5460, P5807, P5997, P6607, P7310, P7476, P8911, P11744	CCM 3730^T^	KACC 21477^T^	CCM 3331	CCM 3308	CCM 160
Summed feature 3^*b*^	58.1	50.6–60.5	56.8	47.9	36.6	62.5	38.9
C_16:0_	23.8	21.7–24.0	21.6	23.3	24.7	15.7	28.1
C_12:0_	7.0	6.4–9.5	8.8	9.9	4.4	1.0	3.4
Summed feature 8^*b*^	4.9	4.7–7.0	5.6	7.3	16.0	2.0	6.1
C_10:0_ 3OH	5.1	4.6–5.9	4.9	5.1	3.5	2.6	4.7
C_10:0_	TR	TR	TR	TR	ND	TR	TR
C_14:0_	TR	TR; except for 1.0 in P7476	TR	TR	2.8	10.0	TR
C_12:0_ 2OH	ND	ND	ND	ND	2.2	ND	2.5
C_12:0_ 3OH	ND	ND	ND	ND	3.2	3.5	ND
iso-C_16:0_	ND	ND	ND	ND	2.0	ND	ND
C_17:0_ cyclo	ND	ND	TR	5.0	2.5	ND	13.1
C_18:1_ *ω*9c	ND	ND; except for 1.8 in P7476	ND	ND	ND	ND	ND
C_18:0_	ND	ND; except for 5.5 in P7476	TR	TR	TR	TR	TR

a Rugamonas rubra CCM 3730^T^, *Rugamonas rivuli* KACC 21477^T^, Chromobacterium violaceum CCM 3331, *Iodobacter fluviatile* CCM 3308, and Janthinobacterium lividum CCM 160. Data were taken from this study using cells grown to the late exponential phase (48 h) on R2A agar medium at 20°C. TR, traces (<1.0%); ND, not detected.

b Summed features are groups of fatty acids that cannot be separated by gas chromatography using the MIDI system. Summed feature 3 contains C_16:1_*ω7c*/C_16:1_*ω6c*; summed feature 8 includes C_18:1_*ω7c*/iso-C_18:1_*ω6c*.

The polar lipid profile of strain P5900^T^ consisted of the major lipids diphosphatidylglycerol, phosphatidylglycerol, and phosphatidylethanolamine. Furthermore, moderate to minor amounts of several unidentified lipids were detected, including one phospholipid (PL), one aminophospholipid (APL), and six lipids without a detectable functional group (L1 to L6) (Fig. S2). This polar lipid profile was much more complex than those reported for Rugamonas aquatica and Rugamonas rivuli (2). For instance, these two reference species did not show the presence of diphosphatidylglycerol or any of the unidentified lipids PL, APL, or L1-6. The quinone system contained exclusively ubiquinone Q-8, which is in line with R. aquatica and R. rivuli. The polyamine pattern was composed of 16.0 μmol (g dry weight)^−1^ putrescine, 4.1 μmol (g dry weight)^−1^ 2-hydroxputrescine, and 0.2 μmol (g dry weight)^−1^ spermidine. This polyamine pattern resembles those of numerous *Betaproteobacteria*, including members of *Oxalobacteraceae*, such as Janthinobacterium lividum (23), Massilia norwichensis (24), Pseudoduganella danionis (25), and Undibacterium pigrum (26).

### Violacein pigment.

In order to clarify the nature of the blue-violet pigment present in *Rugamonas* isolates, we extracted lyophilized cell cultures with an excess of methanol. The obtained extracts were separated by reverse-phase high-performance liquid chromatography (HPLC) in a C_18_ column (see Materials and Methods). For all the analyzed isolates, the extract contained a single pigment, whose retention time, as well as its absorbance spectra, corresponded to an authentic standard of violacein and to the reference violacein-producer *Massilia violaceinigra* CCM 8877^T^. We therefore concluded that all the analyzed *Rugamonas* isolates produce violacein endopigment.

### Phenotypic characteristics.

The set of 11 strains represented aerobic, psychrotolerant, Gram-stain-negative, and nonfermenting rods with long polar flagella; cells occurred singly or in irregular clusters and produced moderately slimy colonies with a dark blue-violet endopigment, and the category of pigment is violacein. All strains grew aerobically on R2A agar plates (Oxoid) and plate count agar (PCA) plates (Oxoid), while negative growth was observed on tryptone soya agar (TSA; Oxoid), brain heart infusion agar (BHI; Oxoid), MacConkey agar (Becton, Dickinson), Mueller-Hinton agar (MHA; Oxoid), or nutrient agar CM03 (Oxoid) at 20°C. Good growth was observed between 1°C and 25°C with optimum temperature between 15°C to 20°C. Negative growth was discovered at 30°C. Cells grew well in the pH range of 6.0 to 9.0, whereas pH 4.0 and pH 10.0 inhibited the growth. Good growth was revealed on R2A medium in the presence of 0.5% NaCl (wt/vol), and growth was mostly weak in the presence of 1% NaCl; the presence of 2% NaCl inhibited the growth. The full phenotypic classification of 11 isolated strains is summarized in the species description below. The tests distinguishing the proposed novel species from other *Rugamonas* spp. as well as phenotypically similar violacein-producing species are shown in Table 7, and the species were differentiated from each other by four to eight tests of various types.

**TABLE 7:** Phenotypic differentiation of *Rugamonas violacea* sp. nov

Test	Result for:									
	1^*a*^^,^^*b*^	2	3	4	5	6	7	8	9	10
Violet pigment	+	−	−	−	+	+	w	+	+	+
Red pigment	−	+	−	−	−	−	−	−	−	−
Growth in 0.5% NaCl	+	−	+	w	w	−	+	+	+	+
Growth on nutrient agar and TSA	−	−	+	−	−	−	+	+	+	+
Oxidase	−	+	+	+	−	−	+	+	+	+
Malonate utilization	+	−	−	+	−	−	+	−	−	+
Hydrolysis of:										
DNA	−	+	+	w	+	−	+	−	+	−
Starch	−	−	+	+	+	+	−	−	−	−
Tween 80	−	−	+	+	+	+	+	+	+	+
API ZYM										
α-chymotrypsin	w	−	−	−	−	w	−	−	w	+
Naphthol-AS-Bl-phosphohydrolase	+	−	+	+	−	+	+	+	+	+

a 1. *Rugamonas violacea* sp. nov., 2. Rugamonas rubra CCM 3730^T^, 3. *Rugamonas aquatica* KACC 21316^T^, 4. *Rugamonas rivuli* KACC 21477^T^, 5. *Massilia atriviolacea* CCM 8999^T^, 6. *Massilia violaceinigra* CCM 8877^T^, 7. Massilia violacea CCM 9083^T^, 8. *Iodobacter fluviatile* CCM 3308, 9. Chromobacterium violaceum CCM 3331, 10. Janthinobacterium lividum CCM 160.

b Data are uniform for all isolates of *R. violacea* sp. nov. +, positive; w, weak; −, negative.

### Screening for antibacterial activity of violacein-pigmented bacteria.

Initially, antibacterial activity was investigated using colony picking method, but the procedure was shown to be unsuitable for slowly growing Antarctic bacteria. Thus, modification combining picking and agar spot tests was applied and all 11 isolates displayed zones of inhibition around the growth spots in any of the target pathogens (Fig. S3). Scored inhibition zones of tested isolates on R2A agar are mentioned in Table S3, and dimensions of the zones varied from 22 mm to 39 mm depending on tested and target bacteria. The formation of clear zones on lawn cultures of target bacteria proved extracellular bactericidal activity of the isolates against pathogens via production of extracellular antibacterial compounds. The production of such compounds was higher and zone-reading was better on R2A agar plates than on PCA plates.

### Conclusions.

The polyphasic approach used for the taxonomic classification of 11 strains using 16S rRNA gene sequencing, whole-genome sequencing, MALDI-TOF MS, ribotyping, rep-PCR, chemotaxonomic analyses (menaquinone, polyamines, polar lipids, and fatty acid methyl ester analysis [FAME]), and extended phenotyping distinguished the set of isolates from their closest relatives. The obtained results proved that the group of violet-pigmented strains isolated from various freshwater sources in Antarctica represents a novel *Rugamonas* species for which the name *Rugamonas violacea* sp. nov. is proposed. The number of isolated strains obtained from the different aquatic localities on James Ross Island indicated the presence of *Rugamonas violacea* as an autochthonous part of the water microbiome in the Antarctic water ecosystem.

## MATERIALS AND METHODS

### Isolation, cultivation, and reference strains.

Sampling was carried out by spreading 50 μl of a water sample with an L loop on the surface of an R2A agar plate, or repeating with 100 μl of water sample if the growth in the first step was inadequate, and cultivating at 15°C for up to 5 days. Individual violet- to dark purple-pigmented colonies were continuously picked out and purified by repeated streaking on R2A medium at 15°C, and the obtained pure cultures were maintained at −70°C until analyzed. Reference strains of the phylogenetic relative Rugamonas rubra CCM 3730^T^, *Rugamonas rivuli* KACC 21477^T^, and *Rugamonas aquatica* KACC 21316^T^ were obtained from the Czech Collection of Microorganisms (CCM) and Korean Agricultural Culture Collection (KACC).

### DNA extraction, 16S rRNA sequencing, and phylogenetic analyses.

DNA for molecular analyses was extracted with FastPrep Lysing Matrix type B and a FastPrep homogenizer (MP Biomedicals, Irvine, CA, USA) and purified with a High Pure PCR template preparation kit (Roche Diagnostics, Mannheim, Germany). The amplification of the partial 16S rRNA gene corresponding to coordinates 8 to 1,542 (Escherichia coli nomenclature) was done by PCR with FastStart PCR Master (Roche Diagnostics) and conserved forward primer pA (5′-AGAGTTTGATCCTGGCTCAG-3′) and reverse primer pH (5′-AAGGAGGTGATCCAGCCGCA-3′) (27), purified using a QIAquick PCR purification kit (Qiagen, Hilden, Germany), and sequenced with a forward primer (5′-GTGGGGAKCRAACAGGATTAG-3′) and a reverse primer (5′-CACATSMTCCMCCRCTTGT-3′) (28). Initial identification of the strains to the genus level was based on pairwise sequence alignment and calculation of similarity values with the algorithm used in the EzBioCloud database (29). 16S rRNA gene sequences obtained from PCR products were aligned with those extracted from whole-genome sequencing (WGS) data using RNAmmer version 1.2 (30). The phylogenetic analyses were performed with the software MEGA X (31). Genetic distances were corrected using Kimura’s two-parameter model (32), and the evolutionary history was inferred using the maximum-likelihood (ML) and neighbor-joining (NJ) methods, using a bootstrap test based on 1,000 replications.

### Genome analysis and calculating average nucleotide identity values.

The *de novo* WGS analysis was performed for the detailed taxonomic characterization of P5900^T^. The whole-genome sequencing and its annotation were carried out in the framework of the Global Catalogue of Microorganisms (GCM) 10K type strain sequencing project as described previously (33, 34). The whole-genome sequence of P5900^T^ (GenBank accession JAEMNU000000000) was compared to those of the related Rugamonas rubra ATCC 43154^T^ (GenBank accession FOTW00000000), *Rugamonas aquatica* GDMCC 1.1643^T^ (GenBank accession WHUG00000000), and *Rugamonas rivuli* GDMCC 1.1685^T^ (GenBank accession WHUF00000000) obtained from the NCBI database. The average nucleotide identity (ANI) values calculation using the OrthoANI algorithm was carried out using the ANI calculator implemented on the EzBioCloud server (35). The dDDH values were calculated using the genome-to-genome distance calculator (GGDC) version 2.1 (36), taking recommended formula 2 into account.

### Bioinformatic analyses.

A genome annotation was added using the NCBI Prokaryotic Genome Annotation Pipeline (PGAP) (37), and an operon prediction was completed using Operon-mapper (38). The functional annotation of the protein-coding genes was performed by assigning clusters of orthologous group categories from the eggNOG database with eggNOG-mapper v.5.0 (39). Prophage DNA was searched with PHASTER (40). The annotated genome sequence was further analyzed for the presence of CRISPR loci using the CRISPRDetect tool (41). Metabolic pathway mapping was done by searching for orthologues in KEGG (42) using BlastKOALA (43), while annotation was completed manually by BLAST searches (44). Motifs were searched using CLC Genomics Workbench v20.0.4. The resistome was predicted with Resistance Gene Identifier (RGI) 5.1.1, using the Comprehensive Antibiotic Resistance Database (CARD) 3.1.1 (45).

### Matrix-assisted laser desorption ionization–time of flight mass spectrometry.

Protein fingerprinting was carried out by means of MALDI-TOF MS using an UltrafleXtreme instrument (Bruker Daltonic), conducted after samples were treated with the ethanol/formic acid/extraction protocol (46). Due to the impossibility of separating the cellular material from its suspension in 70% EtOH by centrifugation, a vacuum evaporator was used for complete liquid removal.

### Further genotypic differentiation of isolates.

The genomic relatedness among isolated strains was studied by rep-PCR analyses with the (GTG)_5_ primer according to Švec et al. (47) and automated ribotyping with the *Eco*RI restriction enzyme using a RiboPrinter microbial characterization system (DuPont Qualicon) in accordance with the protocol provided by the manufacturer. Numerical analysis and dendrogram construction were done using the software BioNumerics 7.1 (Applied Maths). The ribotype patterns were exported into the BioNumerics database using the load samples import script provided by the manufacturer.

### Chemotaxonomic characterization.

Fatty acid methyl ester analysis (FAME) was performed with cells growing on R2A agar (Oxoid) incubated at 20°C ± 2°C for 72 h, as described by Švec et al. (48). Quinones and polar lipids were extracted from freeze-dried biomass grown on R2A medium. Polyamines were extracted as reported by Busse and Auling (23) and analyzed by HPLC, applying the conditions reported by Busse et al. (49). The analysis of quinones and polar lipids was carried out by applying the integrated procedure reported by Tindall (50, 51) and Altenburger et al. (52). For HPLC analyses, the apparatus was used as described by Stolz et al. (53).

### Violacein determination.

To identify the nature of the purple pigment, lyophilized cells of each *Rugamonas* isolate were homogenized by bead-beating in 1 ml of 95% methanol. The extract (100 μl) was analyzed by Agilent-1200 HPLC. The separation was carried out in a reverse-phase column (C_18_ Luna Omega 5 μm Polar, 150 by 2.1 mm; Phenomenex) with 35% methanol and 15% acetonitrile in 0.25 M pyridine as solvent A and 50% methanol in acetonitrile as solvent B. Pigments were eluted with a linear gradient of solvent B (10 to 100% in 30 min) at 40°C and at a 0.8 ml/min flow rate. Violacein was detected at 575 nm using an Agilent 1260 diode-array detector, and the purified violacein from Janthinobacterium lividum served as an authentic standard (Sigma-Aldrich).

### Phenotypic and physiological characterization.

The Gram-staining results of the analyzed strains were confirmed by the KOH lysis test method (54). Growth on several media such as PCA, TSA, nutrient agar, MHA, MacConkey agar, and BHI was evaluated at 20°C. Growth at different temperatures (1, 5, 10, 15, 20, 25, and 30°C) and tolerance to various NaCl concentrations (0.5, 1, 2, and 3% wt/vol) were determined based on cultivation on R2A agar plates for up to 4 days (28). The pH range for growth was tested on R2A agar plates adjusted to pH 4.0 to 10.0 using a buffer system (pH 4.0 to 8.0, 0.1 M KH_2_PO_4_/0.1 M NaOH; pH 9.0 to 10.0, 0.1 M NaHCO_3_/0.1 M Na_2_CO_3_; at intervals of 1 pH unit) for up to 4 days at 20°C (55). The basic phenotyping was performed using conventional tube and plate tests relevant for Gram-negative rods as described previously (28, 56–58). The activities of amylase and protease were tested using R2A agar plates supplemented with appropriate substrates (59). These key tests were inoculated with cells grown at 20°C for 48 h on R2A agar. Additional biotyping with a GEN III MicroPlate identification test kit, using protocol C1 (Biolog) and an API ZYM kit (bioMérieux) according to the manufacturer’s instructions, enabled a comprehensive characterization of isolates. Inoculated kits were incubated at 20°C, and the results were read after 18 h (API ZYM) and 48 h (GEN III MicroPlate). Differences in the antibiotic resistance patterns were tested by the disk diffusion method on R2A agar (Oxoid) for 2 days at 20°C. Twelve antibiotic disks generally used for Gram-negative rods were chosen: ampicillin (10 μg), carbenicillin (100 μg), ceftazidime (10 μg), ciprofloxacin (5 μg), gentamicin (10 μg), chloramphenicol (30 μg), imipenem (10 μg), kanamycin (30 μg), co-trimoxazole (25 μg), piperacillin (30 μg), streptomycin (10 μg), and tetracycline (30 μg) (48, 60, 61). EUCAST/CLSI standards were followed strictly for reading cultivation and inhibition zone diameters (60, 61).

### Screening method for antibacterial activity.

A total of 11 pigmented isolates were tested for their ability to inhibit growth of indicator microorganisms which were obtained from Czech Collection of Microorganisms (CCM; Brno, Czech Republic). The indicator strains of Escherichia coli CCM 3954, E. coli CCM 5172^T^, Staphylococcus aureus CCM 885^T^, S. aureus CCM 3953, S. aureus CCM 2022, and Enterococcus faecalis CCM 7000^T^ were used. Pure colonies of indicator and test bacteria were prepared by streaking a loopful of stock onto agar plates. For the detection of antimicrobial activity, the colony picking method (62) was applied initially and R2A agar and PCA agar were used. Subsequently, the antimicrobial activity testing procedure was modified by application of an agar spot test (63). Briefly, fresh pigmented strains were inoculated as a picking spot on R2A and PCA agar plates and incubated at 20°C for 72 h to allow colonies to develop. Afterwards, the plates were overlaid with 10 ml of 0.7% (wt/vol) LB or BHI agar at 45°C, previously inoculated with 10 μl (10^7^ to 10^9^ CFU ml^−1^) of an overnight culture of the indicator pathogen strain. The plates were incubated aerobically at 37°C and clear zones of inhibition around the spots were examined and scored. All tests were carried out in three independent experiments.

### Data availability.

Accession numbers provided in this article as a result of this study are following: the GenBank/EMBL/DDBJ accession number for the complete 16S rRNA gene sequence of strain P5900^T^ is MT984570. The genome accession number of P5900^T^ in the GCM type strains genome database is GCM60018930. The Whole-Genome Shotgun project of P5900^T^ has the accession no. JAEMNU000000000 at GenBank/EMBL/DDBJ. The GenBank/EMBL/DDBJ accession numbers for the partial 16S rRNA gene sequences of strains P4871, P5042, P5460, P5807, P5997, P6607, P7310, P7476, P8911 and P11744 are MT984571 to MT984580.
