# Supplementary material for: Classification of a Violacein-Producing Psychrophilic Group of Isolates Associated with Freshwater in Antarctica and Description of Rugamonas violacea sp. nov
Source: Microbiol Spectr. 2021 Aug 11;9(1):10.1128/spectrum.00452-21. doi: 10.1128/spectrum.00452-21 (PMC8552646; doi:10.1128/spectrum.00452-21)
Supplement: SUPPLEMENTAL FILE 1 — Supplemental material. Download SPECTRUM00452-21_Supp_1_seq8.pdf, PDF file, 0.5 MB [file spectrum00452-21_supp_1_seq8.pdf]

Classification of a violacein-producing psychrophilic group of isolates associated with freshwater in Antarctica and description of *Rugamonas violacea* sp. nov.

Ivo Sedláček[a,*], Pavla Holochová[a], Roman Sobotka[b], Hans-Jürgen Busse[c], Pavel Švec[a], Stanislava Králová[a], Ondrej Šedo[d], Jan Pilný[b], Eva Staňková[a], Vendula Koublová[a], Karel Sedlář[e]

[a] Department of Experimental Biology, Czech Collection of Microorganisms, Faculty of Science, Masaryk University, Kamenice 5, 625 00 Brno, Czech Republic; ivo@sci.muni.cz (I.S.); pavlah@sci.muni.cz (P.H.); stanci@mail.muni.cz (S.K.); evickakroupova@seznam.cz (E.S.); 436839@mail.muni.cz (V.K.); mpavel@sci.muni.cz (P.Š.)

[b] Centrum Algatech, MBÚ AV ČR, Novohradská 237 – Opatovický mlýn, 379 01 Třeboň, Czech Republic; Sobotka@alga.cz (R.S.); pilny@alga.cz (J.P.)

[c] Institut für Mikrobiologie, Veterinärmedizinische Universität Wien, Veterinärplatz 1, A-1210 Wien, Austria; Hans-Juergen.Busse@vetmeduni.ac.at

[d] Central European Institute of Technology, Masaryk University, Kamenice 5, 625 00 Brno, Czech Republic; sedo@post.cz

[e] Department of Biomedical Engineering, Faculty of Electrical Engineering and Communication, Brno University of Technology, Technická 12, 616 00 Brno, Czech Republic; sedlar@vut.cz

***Correspondence**: ivo@sci.muni.cz; Tel.: +420-549-496-922

Supplementary materials

Table S1a. Clusters of orthologous groups of *Rugamonas violacea* P5900^T

COG class	Description	Gene count	Percentage
A	RNA processing and modification	1	0.02
B	Chromatin structure and dynamics	4	0.07
C	Energy production and conversion	284	4.89
D	Cell cycle control, cell division, chromosome partitioning	68	1.17
E	Amino Acid transport and metabolism	416	7.17
F	Nucleotide transport and metabolism	118	2.03
G	Carbohydrate transport and metabolism	172	2.96
H	Coenzyme transport and metabolism	199	3.43
I	Lipid transport and metabolism	186	3.20
J	Translation, ribosomal function and biogenesis	200	3.45
K	Transcription	428	7.37
L	Replication, recombination and repair	163	2.81
M	Cell wall/membrane/envelope biogenesis	357	6.15
N	Cell motility	252	4.34
O	Posttranslational modification, protein turnover, chaperones	169	2.91
P	Inorganic ion transport and metabolism	248	4.27
Q	Secondary metabolites biosynthesis, transport and catabolism	118	2.03
S	Function Unknown	1017	17.52
T	Signal transduction mechanisms	324	5.58
U	Intracellular trafficking, secretion, and vesicular transport	142	2.45
V	Defense mechanisms	87	1.50
	COG unknown	852	14.68

Table S1b. CRISPR arrays in the *Rugamonas violaea* P5900^T genome

contig	Start	End	Len.	DR consensus	DR len.	No. of spac.
83	147275	147533	259	CGGCACGCCCGAGTAGGCGACG GCCGGCACGCCGGA	36	3
102	52868	56743	3876	GTCGCGTCCCGCGTGGGCGCGT GGATTGAAAC	32	58
102	65844	68320	2477	GTCGCGCTCCGCGTGGGCGCGT GGATTGAAAC	32	37

Table S2. Antibiotic resistance genes in the *Rugamonas violacea* P5900^{T} genome

Accession	Best hit to Antibiotic Resistance Ontology (ARO)	Sequence Similarity	ARO	Drug Class	Resistance Mechanism	AMR Gene Family
MBJ7310140.1	*Escherichia coli* EF-Tu mutants conferring resistance to Pulvomycin	78.99	3003369	elfamycin antibiotic	antibiotic target alteration	elfamycin resistant EF-Tu
MBJ7310207.1	*Escherichia coli* EF-Tu mutants conferring resistance to Pulvomycin	78.99	3003369	elfamycin antibiotic	antibiotic target alteration	elfamycin resistant EF-Tu
MBJ7313916.1	adeF	69.46	3000777	fluoroquinolone antibiotic; tetracycline antibiotic	antibiotic efflux	resistance-nodulation-cell division (RND) antibiotic efflux pump
MBJ7311516.1	LRA-10	69.15	3002489	cephalosporin; penam	antibiotic inactivation	class C LRA beta-lactamase
MBJ7314050.1	acrB	66.22	3000216	fluoroquinolone antibiotic; cephalosporin; glycylcycline; penam; tetracycline antibiotic; rifamycin antibiotic; phenicol antibiotic; triclosan	antibiotic efflux	resistance-nodulation-cell division (RND) antibiotic efflux pump
MBJ7309657.1	kdpE	65.93	3003841	aminoglycoside antibiotic	antibiotic efflux	kdpDE
MBJ7309473.1	*Pseudomonas aeruginosa* soxR	63.12	3004107	fluoroquinolone antibiotic; cephalosporin; glycylcycline; penam; tetracycline antibiotic; acridine dye; rifamycin antibiotic; phenicol antibiotic; triclosan	antibiotic target alteration; antibiotic efflux	ATP-binding cassette (ABC) antibiotic efflux pump; major facilitator superfamily (MFS) antibiotic efflux pump; resistance-nodulation-cell division (RND) antibiotic efflux pump
MBJ7312309.1	MuxB	60.54	3004074	macrolide antibiotic; monobactam; tetracycline antibiotic; aminocoumarin antibiotic	antibiotic efflux	resistance-nodulation-cell division (RND) antibiotic efflux pump
MBJ7311207.1	LRA-1	60.42	3002482	cephalosporin; penam	antibiotic inactivation	class A LRA beta-lactamase
MBJ7313189.1	qacL	60.19	3005098		antibiotic efflux	small multidrug resistance (SMR) antibiotic efflux pump
MBJ7310206.1	*Staphylococcus aureus* fusA with mutation conferring resistance to fusidic acid	59.17	3003735	fusidic acid	antibiotic target alteration	antibiotic resistant fusA
MBJ7308813.1	*Staphylococcus aureus* fusA with mutation conferring resistance to fusidic acid				antibiotic target alteration	antibiotic resistant fusA
MBJ7313357.1	MSI-OXA				antibiotic inactivation	MSI-OXA family beta-lactamase

Table S3. Antibacterial activity of *Rugamonas violacea* sp. nov. strains.

Target bacteria	Tested bacteria - clear zone diameter (mm) on R2A medium										
	P4871	P5042	P5460	P5807	P5900^T	P5997	P6607	P7310	P7476	P8911	P11744
E. coli CCM 3954	27	22	23	25	28	27	26	32	29	29	30
E. coli CCM 5172^T	32	31	31	30	32	27	27	36	37	32	35
S. aureus CCM 885^T	39	34	33	28	29	23	34	36	30	34	27
S. aureus CCM 3953	30	27	21	30	32	23	25	36	32	30	32
S. aureus CCM 2022	32	27	29	22	26	29	25	31	30	32	29
E. faecalis CCM 7000^T	28	28	26	26	32	30	23	32	31	32	29

All these isolates could be identified as producing extracellular antibacterial compounds.

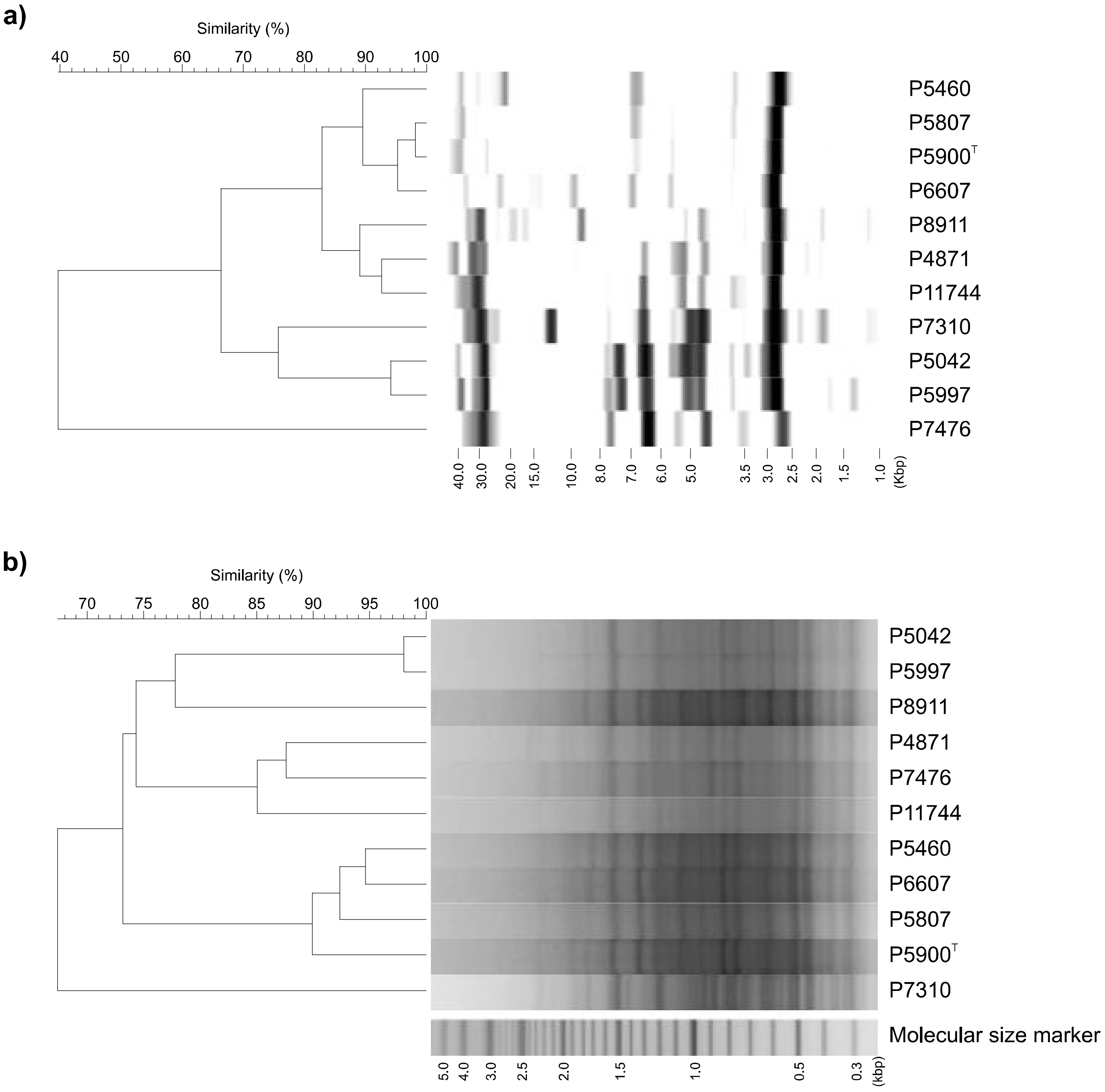

Fig. S1. Dendrograms based on cluster analysis of **a)** *Eco*RI ribotype patterns obtained using the RiboPrinter identification system and **b)** $(GTG)_5$-PCR fingerprints from *Rugamonas violacea* sp. nov. strains. The dendrograms were calculated with Pearson's correlation coefficients with UPGMA clustering method (*r*, expressed as percentage similarity values).

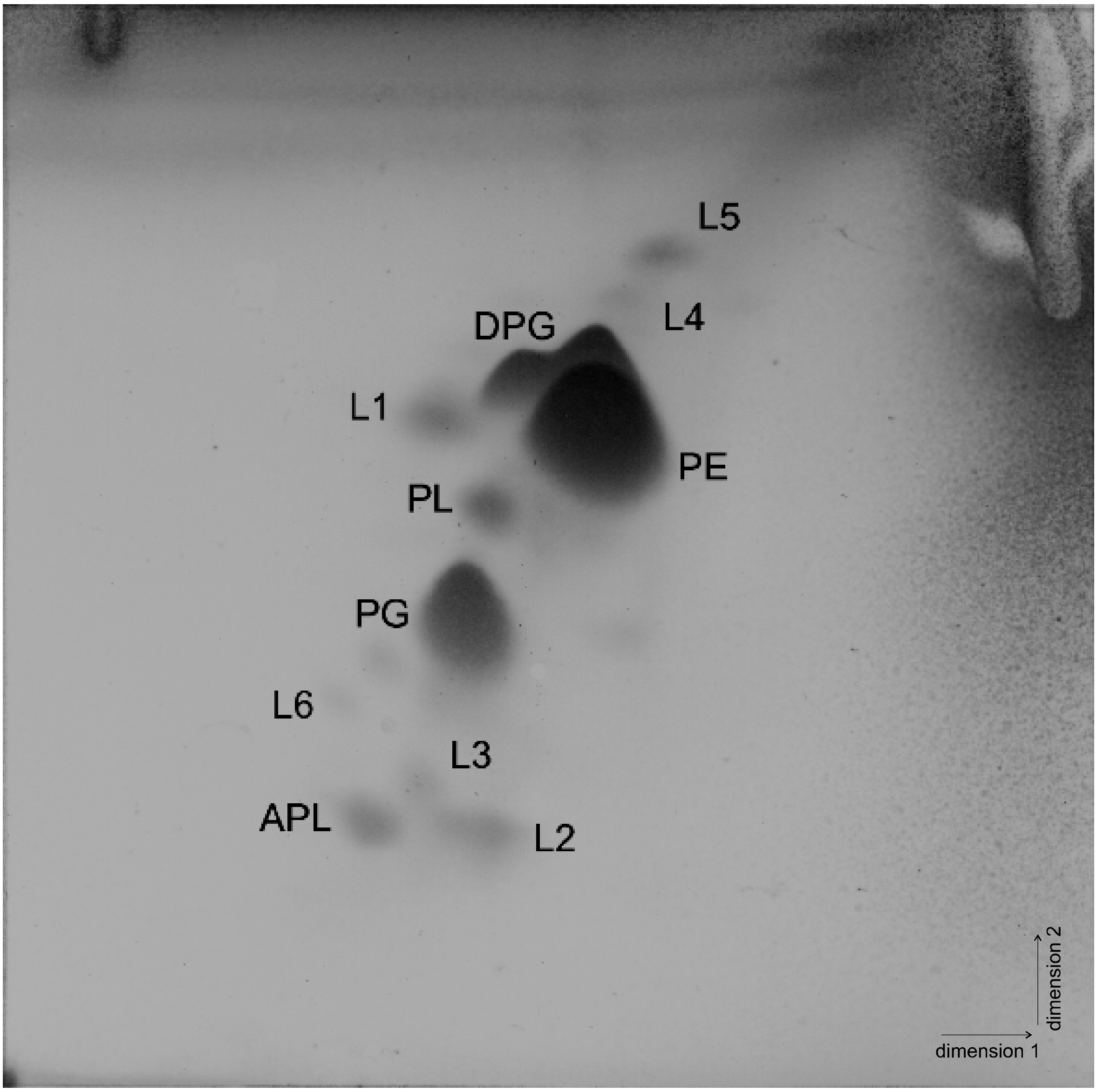

Fig. S2. Polar lipid profile of strain CCM 8940[T] after two-dimensional thin layer chromatography, detection with 5 % ethanolic molybdatophosphoric acid at 140 °C. Abbreviations: PE, phosphatidylethanolamine; PG, phosphatidylglycerol; DPG, diphosphatidylglycerol; APL, unidentified aminophospholipid; PL, unidentified phospholipid; L1-6, unidentified polar lipids lacking a functional group.

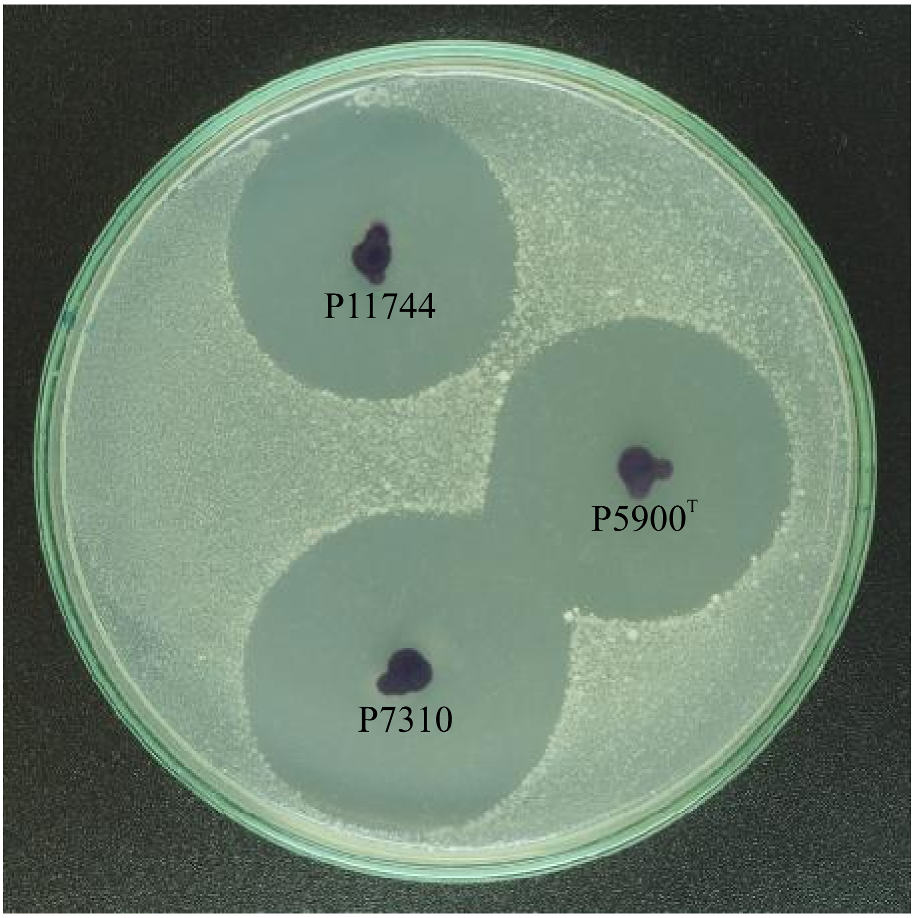

Fig. S3. Zones of inhibition among *R. violacea* representatives and indicator strain *S. aureus* CCM 3953. Clear zones are the result of test bacteria inhibiting the growth of target strain.
